# Impact of Familiarity and Green Image on Satisfaction and Loyalty Among Young Green Hotels’ Guests – A Developing Country’s Perspective

**DOI:** 10.3389/fpsyg.2022.899118

**Published:** 2022-05-20

**Authors:** Lei Wang, Qi Zhang, Philip Pong Weng Wong

**Affiliations:** ^1^Department of Hospitality and Tourism, School of Management, Xuzhou University of Technology, Xuzhou, China; ^2^School of Management, Xuzhou University of Technology, Xuzhou, China; ^3^School of Hospitality, Sunway University, Selangor, Malaysia

**Keywords:** familiarity, green image, satisfaction, loyalty, green hotel patronage

## Abstract

Familiarity and green image influencing travelers’ green hotel patronage have often been overlooked in previous research. The aim of this study was to examine the relationship of the two dimensions of familiarity and the two dimensions of image with the overall image, satisfaction, and loyalty. The theoretical research model used in this study was based on the stimulus–organism–response framework. The research design for this study employed a quantitative approach using a purposive sampling method involving 488 student responses. The tests of the proposed hypotheses were conducted using SPSS and AMOS. The results of the study showed that experiential familiarity and informational familiarity positively influenced cognitive image and affective image, respectively, while cognitive image had a positive influence on the affective image. In addition, cognitive and affective images have a positive influence on the overall image, and the overall image is shown to have a positive impact on satisfaction and loyalty. Finally, satisfaction was found to have a positive influence on loyalty, while satisfaction plays a partial meditation role in the relationship between overall image and loyalty.

## Introduction

The tourism industry has been traditionally perceived as a clean and environmentally friendly industry (Wang, 2022). However, recent studies have accused the hospitality industry to be a major contributor to environmental pollution (Al-Aomar and Hussain, 2017; Nimri et al., 2020). Approximately, 75% of the negative environmental impact caused by the hospitality industry may be attributed to over-consumption of natural resources (e.g., water, food, energy, and non-durable goods) (Sadiq et al., 2022), One positive result of this negative impact of tourism is a growth in the green awareness of environmental issues by leisure travelers (Wang, 2022). Due to the increase in green awareness, 22% of travelers are now deliberately looking for green information to assist them in making reservations for green hotels. It is estimated that 40% of travelers are willing to pay an extra 4–6% for green hotels (Wang et al., 2020a), and 61% of travelers prefer staying in green hotels (Sadiq et al., 2022). Although more consumers are demanding pro-environmentally facilities and services in hotels (Wang et al., 2021a) and the popularity of green hotels has been on the rise, the actual patronage of green hotels is still comparatively low (Sadiq et al., 2022). This highlights a gap between environmental attitude and behavior in the green hotel literature (Wang et al., 2020b).

Many researchers have called for a deeper investigation of the reason for the attitude–behavior gap (Wang et al., 2021b). Some studies focused on the environmental behavior of specific tourists, such as those who booked eco-cruises (Han et al., 2019), preferred green foods (Yogananda and Nair, 2019), and adopted green washing practices (Chen et al., 2019), rather than looking at tourists’ patronage patterns of green hotels. Hence, it can be difficult to generalize the results of these studies to green hotel literature (Nimri et al., 2020). Previous studies on green hotel selection have focused on the relationship between travelers’ attitudes and intention/behavior and their green hotel patronage (Wang, 2022).

Unfortunately, most travelers are not very familiar with the advantages and the operating mechanisms of green hotels (Wang et al., 2018). Most travelers have only heard about the concept of green hotels (Wang et al., 2018) and usually select traditional hotels when they are traveling, as they are still unsure of what green hotels have to offer (Choi et al., 2015). This type of perception is particularly widespread in some developing countries (Arun et al., 2021). Wang and Wong (2021) stressed that although the concept of green hotels has been extensively explored from the western perspective, it still lacks a standardized definition and a coherent foundation. The literature on consumer patronage of green hotels is currently dominated by research in the developed nations without much consideration of the contributions coming from the consumers of the developing countries (Arun et al., 2021).

A destination’s image is often formed by a traveler’s exposure to various information sources and their prior knowledge and experience of the destination (Casali et al., 2020). Studies have shown that the green image of a hotel may lead to stronger customer loyalty and will likely result in positive word-of-mouth about the green hotel (Wang et al., 2018). However, there is a dearth of research on the relationship between the green image and loyalty in developing nations (Wu et al., 2016, 2019; Çavusoglu et al., 2020) and how this relationship is mediated by satisfaction (Wang et al., 2018; Merli et al., 2019; Çavusoglu et al., 2020). Familiarity has been shown to affect the destination image in previous studies (Casali et al., 2020), but researchers have begun to criticize the measurement of the single-dimension construct of destination familiarity which is deemed to be inappropriate in the temporal tourism literature (Jebbouri et al., 2021), as destination familiarity can be increased by both direct experience and indirect experience (Kim et al., 2019; Stylidis et al., 2020). Very few studies have investigated the relationship between familiarity and green image in green hotel selection in developing countries. Hence, this Study attempts to fill these gaps by predicting travelers’ loyalty toward green hotels by incorporating familiarity and image in the stimulus–organism–response (S-O-R) framework. Therefore, this study will contribute to the body of knowledge on the influence of experiential familiarity, informational familiarity, cognitive image, affective image, overall image, and satisfaction on green hotels’ loyalty in China.

## Literature Review

### Underpinning Theory of Study

**FIGURE 1:**
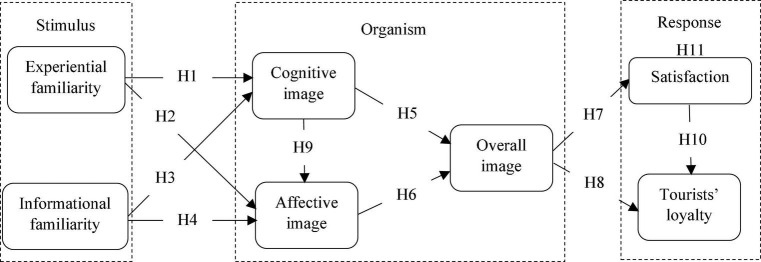
Conceptual research model.

This study incorporated the S-O-R framework (Mehrabian and Russell, 1974) in its conceptual model (Figure 1). The S-O-R framework is one of the most popular behavioral frameworks in the field of marketing research (Wang et al., 2018) that is frequently used to explain the relationship between the stimuli individuals receive, the internal evaluations generated, and the resulting attitude or response (Mehrabian and Russell, 1974). The stimuli derived from the companies were described as a set of attributes that influences consumers’ perceptions (Su et al., 2016), while the organism is the intervening internal process between the stimuli and the response of consumers (Wang et al., 2018), and this process consists of physiological and perceptual feelings, and thinking activities (Bagozzi, 1986). The response is the final outcome or reaction toward the company, which may include psychological reactions such as attitude and intention (Bagozzi, 1986). The S-O-R framework provides the means to explore the effect of other contextual variables which provide an explanation for certain environmental behaviors (Wang et al., 2018). In this study, the experiential familiarity and informational familiarity were considered as stimuli that elicit the travelers’ internal evaluations of the cognitive image, affective image, and overall image (i.e., organism), which can have an impact on their satisfaction and loyalty (i.e., response) toward green hotels.

### Familiarity, Cognitive Image, and Affective Image

Familiarity is a crucial factor that influences a traveler’s destination image, perceptions, destination selection, and behavior in the future (Bianchi et al., 2017; Casali et al., 2020). However, very few studies have investigated the extent to which familiarity affects travelers’ perceptions and behavior with regard to a tourist destination (Kim et al., 2019). Destination familiarity has frequently been described as a single-dimension construct that is measured via past experience in the destination (Jebbouri et al., 2021). Past studies have often used destination familiarity to compare the first visit and revisit experiences (Prentice, 2006). Familiarity is often used to explain the differences in travel behavior between visitors and non-visitors in a given destination (Jebbouri et al., 2021). In other words, destination familiarity is increased by actual purchase and usage (Alcocer and Ruiz, 2019), and thus, direct experience can influence the development of a positive destination image (Stylidis et al., 2020). Kim et al. (2019) described experiential familiarity as the level of awareness or knowledge of past travel experiences, and familiarity can reduce risk perceptions among travelers and increase confidence in their destination choice (Lepp and Gibson, 2003). Hence, experiential familiarity is a key determinant of the cognitive and affective components of a destination’s image (Casali et al., 2020; Stylidis et al., 2020).

However, many researchers have expressed their concern about using the single-dimension construct measurement of familiarity which they feel is not appropriate in the temporal tourism environment (Jebbouri et al., 2021; Ju et al., 2021). Kim et al. (2019) argued that destination familiarity can be enhanced not only by direct experience, but also by indirect experience without actual travel experience. For example, travelers can gain a certain level of familiarity from information obtained through their contact with other people, travel guides, social media, and education (Gursoy, 2011). Hence, Soliman (2019) described informational familiarity refers to the scope of information sources utilized, operationalized as singular or varied sources. These sources include indirect resources such as books, advertisements, movies, user-generated content, brochures, and websites (Kim et al., 2018; Stylidis et al., 2020). Travelers who possess a high level of external information about a destination tend to depend more on their personal knowledge rather than on other sources (Stylidis et al., 2020). On the other hand, potential travelers with low familiarity tend to rely more on external information sources to assist them in their destination selection (Murphy et al., 2007). Therefore, destination familiarity can be categorized under experiential familiarity and informational familiarity (Kim et al., 2019; Jebbouri et al., 2021).

Earlier studies show how both experiential and informational familiarity can significantly influence travelers’ cognitive and affective image. Manyiwa et al. (2018) argued that because familiarity is enhanced by frequent visits or a period of residence, the cognitive and affective images are more likely to be shaped by experiences for residents and information for travelers. Jebbouri et al. (2021) found that both experiential and informational familiarity have a positive influence on tourists’ cognitive and affective image, leading to greater satisfaction and loyalty. Smith et al. (2015) found that familiarity positively influences the cognitive and affective components of the image. Meanwhile, Soliman (2019) discovered a positive relationship between experiential and informational familiarity and cognitive and affective image. In addition, Baloglu and McCleary (1999) posited that destination image can be affected by stimuli factors, which involve previous experiences and information sources (e.g., word-of-mouth or personal contact with others). Based on the above-mentioned findings, the following hypotheses were proposed for testing:

H1: There is a positive relationship between experiential familiarity and cognitive image.

H2: There is a positive relationship between experiential familiarity and affective image.

H3: There is a positive relationship between informational familiarity and cognitive image.

H4: There is a positive relationship between informational familiarity and affective image.

### Cognitive Image, Affective Image, Overall Image, Satisfaction, and Loyalty

Destination image is among the main determinants that play a significant role in influencing tourist visit and revisit intention (Melo et al., 2017; Wang et al., 2021c), and satisfaction and loyalty (Wang et al., 2018; Jebbouri et al., 2021). However, there has been inadequate research on the influence of destination image on the selection of green hotels (Wang et al., 2018), and there is no consensus on the dimensions of destination image (Pektaş et al., 2019). According to Ragab et al. (2019), destination image can be defined as “a set of feelings, emotions, and attitudes that tourists hold regarding the destination, or a traveler’s overall perception about a destination, and it is claimed that it considerably influences his/her travel decision” (Baloglu and McCleary, 1999). Thus, destination image can be considered a multidimensional construct which is the sum of perceptions, impressions, and beliefs travelers have of the various attributes, aspects, and activities of a destination (Casali et al., 2020). Most previous researchers measure the overall destination image by using the cognitive image, affective image, and conative image components (Králiková et al., 2020; Jebbouri et al., 2021).

According to Jebbouri et al. (2021), the overall image is composed of the cognitive image (which consists of beliefs, perception, and knowledge of a destination’s characteristics or attributes), (Soliman, 2019); affective image (made up of an individual’s feelings or emotional responses toward this destination) (Kim et al., 2019); and the conative image is derived from the cognitive and affective image which leads to actual behavior (Králiková et al., 2020). Many researchers recently have argued that there is a direct positive relationship between conative image, cognitive image, and affective image (Stylos et al., 2017; Stylidis et al., 2020). Therefore, both cognitive and affective images are the best predictors of destination selection (Králiková et al., 2020). A destination’s overall image is composed of the cognitive image, which consists of the physical, functional, and tangible attributes, (Casali et al., 2020) and the affective image, which is associated with feelings or emotional responses about a specific tourist destination (Kim et al., 2019).

According to Chi et al. (2020), the more unique and favorable images the travelers hold in their memories, the stronger the connection a traveler has with the destination. Certain studies confirmed that destination images are widely used in promotional materials to foster awareness of the attributes that set them apart from competitors. Wang et al. (2021c) found that cognitive and affective images positively influence Chinese students’ perceptions toward visiting western countries as their travel destinations. Casali et al. (2020) discovered that both cognitive and affective images displayed a positive influence on the overall image, and subsequently on satisfaction and loyalty. Likewise, Stylidis et al. (2020) proposed that the overall image consisting of cognitive and affective images positively influences travelers’ destination loyalty. Accordingly, the overall image of travelers’ destination can be influenced by their image formation, leading to decision-making and purchasing behavior, such as destination selection (Casali et al., 2020), and it should be considered as the most important factor impacting travelers’ satisfaction and loyalty with the given destination (Králiková et al., 2020). Thus, the following hypotheses were proposed:

H5: There is a positive relationship between cognitive image and overall image.

H6: There is a positive relationship between affective image and overall image.

H7: There is a positive relationship between overall image and satisfaction.

H8: There is a positive relationship between overall image and loyalty.

According to Alcocer and Ruiz (2019), previous studies on destination image mainly focused on cognitive image. Very few studies actually examined the interrelationship between cognitive and affective images (Kim et al., 2019), but the vast majority of studies supported the sequential development of the affective images from cognitive images (Kim et al., 2019; Stylidis et al., 2020), as travelers’ affective evaluations of a destination depend on their knowledge of that destination (Kim et al., 2019). Basically, this means that cognitive evaluation provides a basis for an overall impression of a given destination and therefore should occur prior to the development of an affective image (Kim et al., 2019). However, in recent years, the specific direction of the relationship between cognitive and affective images has been questioned by some current researchers (Kim et al., 2019). Certain attitude theory researchers have argued that the relationship between cognition and emotion in attitude formation is bidirectional (Kim et al., 2019), even though most previous studies showed that cognitive image is an antecedent to the development of a destination’s affective image (Lee et al., 2010; Alcocer and Ruiz, 2019). For example, Alcocer and Ruiz (2019) confirmed that cognitive image positively influenced affective image toward cultural destinations, which was in line with the study by Kim et al. (2019). Another study by Stylidis et al. (2020) highlighted the high explanatory power of cognitive image as a determinant of affective image. These results support the findings of Baloglu and McCleary (1999) which indicated that information and knowledge acquired from direct and indirect sources of a destination are first formed and interpreted which can then influence the travelers’ emotional status. Thus, the following hypothesis is proposed:

H9: There is a positive relationship between cognitive image and affective image.

### Satisfaction and Tourist Loyalty

The travelers’ satisfaction level with a destination is based on their interactions with service providers, and it can be used to predict future behaviors, such as revisit intention (Jebbouri et al., 2021). It is also an important factor for the successful marketing and promotion of a tourism destination because it influences the tourist’s destination selection, duration of stay, revisit intention, and recommendation to others (Ragab et al., 2019). Therefore, satisfaction can be understood as a traveler’s cognitive and affective state, which derives from his/her experience with the destination which can then lead to destination loyalty (Melo et al., 2017). Hence, tourist satisfaction can be defined as a traveler’s cognitive–affective state derived from the ability of the touristic destination to satisfy his/her needs, desires, and expectations (Ragab et al., 2019). Tourists’ satisfaction should be the basic element used to evaluate the destination performance (Agyeiwaah et al., 2016), which leads to certain indicators of loyalty (e.g., visit/revisit intention, recommend to others, and positive word-of-mouth) (Melo et al., 2017; Králiková et al., 2020). These findings are in line with the study conducted by Jebbouri et al. (2021), who found that satisfaction positively influenced tourists’ loyalty. Hence, the following hypotheses are proposed:

H10: There is a positive relationship between satisfaction and loyalty.

H11: Satisfaction mediates the relationship between overall image and loyalty.

## Materials and Methods

### Data Collection

A non-probability purposive sampling method was adopted in this study because it allows the researchers to manipulate self-judgment in choosing cases that would best fit and allow research questions that can be answered to meet the research objectives (Neuman, 2002). University students were selected for the sample based on the following reasons: (i) the Chinese younger generations have shown to possess robust future purchasing power (Wang et al., 2021a); (ii) the students provide an accurate representation of young vacation seekers (Wang et al., 2021c), as the 25- to 30-year-old age bracket was the dominant traditional hotels’ market segment and the below 24-year-old age bracket was the main homestay market (Wang et al., 2021a); and (iii) university students are generally more concerned about environmental issues and can provide more meaningful insights into green studies (Varah et al., 2021). Data were collected from six universities in Xuzhou, Jiangsu, China, from 1st December to 31st December 2021, as the undergraduate student population in Jiangsu exceeded 1.1 million and ranked third-highest in China, where nearly one-fifth of them is located in Xuzhou (Wang et al., 2021a).

Questionnaires were allocated to university lecturers, assistant professors, and associate professors who distributed them to their students. The students were from various departments (e.g., hospitality and tourism, marketing, education, English language, economics, etc.). All questionnaires were distributed via an online system (i.e., WeChat QR code linking with Chinese electronic survey platform^1^) who completed them in the classroom, and participation was voluntary and gift-compensated. The questionnaire items were translated using the back-translation method to ensure translation accuracy. A total of 488 responses were collected, which exceeded the recommended sample size of 384 based on Cochran’s formula (Cochran, 2007). A pilot test was performed with a total of 40 respondents to ensure instrument reliability and validity and to prevent potential data quality issues.

### Measures

The research instrument used was a self-administered and close-ended four-section questionnaire with validated measurement scales that were adopted from previous studies. The first section measured experiential familiarity and informational familiarity. Three items measuring experiential familiarity were adapted from Chi et al. (2020), and five items evaluating informational familiarity were adapted from Soliman (2019). The second section consists of items used to measure a cognitive image, affective image, and overall image. Four items that were used to measure cognitive image were adapted from Soliman (2019), four items used to evaluate affective image were adapted from Ragab et al. (2019), and four items used to measure the overall image were adapted from Chi et al. (2020). The third section included items that were used to assess satisfaction and loyalty. Three items evaluating satisfaction were adapted from Jebbouri et al. (2021), and four items related to loyalty were adapted from Chi et al. (2020). The last section included questions that were used to obtain demographic data. All of the measurement items were evaluated using a five-point Likert scale ranging from “strongly disagree” to “strongly agree,” as it can obtain higher reliability coefficients compared with a seven-point Likert scale (Saleh and Ryan, 1991; Dawes, 2008).

### Common Method Bias (CMB) Issues

To mitigate potential CMB issues, the questionnaire items were reviewed by field experts to minimize respondents’ misunderstanding of the questions, respondents were fairly well represented from different age and gender groups to reduce CMB impact from homogenous issues, and measures using multiple scale types, including bipolar, semantic, Likert, and differential scales, were employed. To detect CMB (Podsakoff et al., 2003), a latent variable was included in the model by connecting it to all observable factors, and the standardized regression evaluated the new model before comparing it with the original model which showed similar results after comparison. Lastly, Harman’s single factor test was used to test whether CMB affected the results. The test results showed an exploratory factor analysis with a single factor accounting for 47.313% of the variance, which is less than the 50% benchmark value, indicating CMB is not a pervasive issue for this study.

## Data Analysis and Results

### Descriptive Analysis

The SPSS version 19 was employed for the descriptive statistics, and Table 1 presents the profiles and characteristics of the respondents. Normality of data can be assumed when Skewness ranges from −2 to + 2 and kurtosis ranges from −7 to + 7 (Byrne, 2016). The results showed that the normality of data was exhibited, as the skewness values were between −1.857 and −0.022, and the kurtosis values were between −0.646 and + 3.495. The Kaiser–Meyer–Olkin and Bartlett’s test of sphericity indicated sampling adequacy with 0.933 and a *p*-value below 0.001.

**TABLE 1:** Sample characteristics (*N* = 488).

Constructs	Characteristics	Frequency	Percentage (%)
Gender	Male Female	276 212	56.6 43.4
Age	Below 18 18 19 20 21 22 23 24 Above 24	8 91 93 162 92 24 12 3 3	1.6 18.6 19.1 33.2 18.9 4.9 2.5 0.6 0.6
Education level	3-years diploma 4-years bachelor Master and above	3 476 9	0.6 97.5 1.8
Seniority level	Fresh Sophomore Junior Senior Other	176 102 162 44 4	36.1 20.9 33.2 9.0 0.8

### Measurement Model Test

To achieve internal reliability, a Cronbach’s alpha value of 0.7 or more should be obtained (Hair et al., 2010). The results of a reliability test showed that the Cronbach’s alpha values of all constructs were greater than 0.7 (Table 2). In the measurement model, composite reliability (CR) should be more than 0.7 and the average variance extracted (AVE) greater than 0.5 threshold (Hair et al., 2010). After dropping off the low factor loadings and cross-loading items (i.e., cognitive image 2, affective image 4, and loyalty 1), convergent validity was established (Table 2). The discriminant validity was assessed by considering the maximum shared squared variance (MSV) and the average shared square variance (ASV). AVE should be higher than MSV and ASV (Hair et al., 2010), and the correlation between each construct should be less than 0.9 (Meyers et al., 2006). As shown in Table 3, the results indicated that discriminant validity was achieved. The measurement model fit indices show that CMIN = 731.164, DF = 231, CMIN/DF = 3.165 < 5, *p* < 0.001, NFI = 0.936 > 0.9, RFI = 0.923 > 0.9, IFI = 0.955 > 0.9, TLI = 0.946 > 0.9, CFI = 0.955 > 0.9, GFI = 0.883, AFGI = 0.847 > 0.8, PGFI = 0.68 > 0.5, PNFI = 0.783 > 0.5, PCFI = 0.799 > 0.5, PRATIO = 0.837, RMSEA = 0.067 < 0.08, and SRMR = 0.038 < 0.08. According to Kline (2015), CMIN/DF value below 3 indicates an acceptable fit, and a value below 5 also means a reasonable fit (Marsh and Hocevar, 1985). RMSEA value lower than 0.08 is considered good, but a value between 0.01 and 0.1 is considered a moderate fit (Meyers et al., 2006; Hooper et al., 2008). According to Ho (2006), there should be at least three indices meeting the threshold values to make the model fit. Considering that the values of the rest of the indices mostly exceed the threshold value, the overall goodness-of-fit indices indicated an acceptable measurement model fit.

**TABLE 2:** Construct validity.

Constructs (Cronbach’s Alpha)	Items	Item loadings	Composite reliability	Average variance extracted
Experiential familiarity (α = 0.956)	1. Compared to an average person, I am very familiar with green hotels 2. Compared to my friends, I am very familiar with green hotels 3. Compared to people who travel a lot, I am very familiar with green hotels	0.955 0.958 0.902	0.957	0.881
Informational familiarity (α = 0.822)	I obtain formation about green hotel from: 1. Brochures and pamphlets 2. Green hotels’ official website 3. Friends and relatives’ interactions 4. Newspaper and magazines 5. Travel guidebook and travel agency	0.684 0.745 0.775 0.617 0.713	0.834	0.503
Cognitive image (α = 0.968)	1. Green hotel has a good quality of life 2. Green hotels are clean accommodation (delete) 3. Green hotel has a good name and reputation 4. Green hotel has a good value for money	0.947 N/A 0.957 0.958	0.968	0.91
Affective image (α = 0.934)	Green hotel is: 1. Pleasant accommodation 2. Relaxing accommodation 3. Arousing accommodation 4. Exciting accommodation (delete)	0.938 0.949 0.853 N/A	0.939	0.836
Overall image (α = 0.908)	1. Green hotel fits my personality 2. My friends will think highly of me if I patronize green hotel 3. The image of green hotel is consistent with my own self-image 4. Patronizing green hotel reflects who I am	0.907 0.889 0.844 0.755	0.913	0.724
Satisfaction (α = 0.803)	1. I am happy with my decision to patronize green hotel 2. I am pleased to have stayed in green hotel 3. I think I did the right thing patronizing green hotel	0.689 0.828 0.789	0.814	0.594
Tourist loyalty (α = 0.933)	1. I consider myself a loyal guest to green hotels (delete) 2. Green hotel would be my first choice of a travel accommodation 3. I will patronize green hotel instead of traditional hotel if they are as good as similar 4. I would advise other people to patronize green hotel	N/A 0.891 0.909 0.925	0.934	0.825

**TABLE 3:** Discriminate validity.

Construct	AVE	MSV	ASV	1	2	3	4	5	6	7
1. Satisfaction	0.594	0.590	0.373	0.771						
2. Experiential familiarity	0.881	0.590	0.354	0.768	0.939					
3. Informational familiarity	0.503	0.370	0.232	0.463	0.405	0.709				
4. Cognitive image	0.910	0.552	0.369	0.576	0.513	0.434	0.954			
5. Affective image	0.836	0.630	0.395	0.586	0.545	0.471	0.717	0.914		
6. Overall image	0.724	0.461	0.351	0.512	0.536	0.608	0.603	0.603	0.851	
7. Tourist loyalty	0.825	0.630	0.483	0.706	0.725	0.483	0.743	0.794	0.679	0.908

### Structural Model Estimation

**FIGURE 2:**
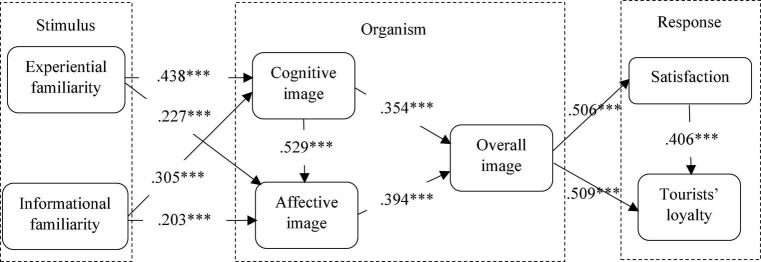
The structural model outcomes. ^***^ denotes *p*-value < 0.001.

Having achieved an acceptable measurement model fit, SEM was performed to test the hypotheses of this study. In the model fit summary, CMIN = 1280.806, DF = 240, CMIN/DF = 5.337, GFI = 0.839, AGFI = 0.799 ≈ 0.8, PGFI = 0.671 > 0.5, IFI = 0.907 > 0.8, CFI = 0.906 > 0.8, PNFI = 0.772 > 0.5, PCFI = 0.788 > 0.5, PRATIO = 0.87, and RMSEA = 0.094 < 1. Again, it is required that the values of at least three indices meet the threshold values to make the structural model fit (Ho, 2006). The model fit for the structural model of this study was achieved based on the results of the SEM conducted as illustrated in Figure 2 and Table 4.

**TABLE 4:** Regression results of structural model.

Hypothesis	Parameter	Estimate	*p*-value	C.R.	Decision
H1	Experiential familiarity → cognitive image	0.438	***^c^ (3.61e-21)^d^	10.405	Supported
H2	Experiential familiarity → affective image	0.227	*** (1.69e-8)	5.84	Supported
H3	Informational familiarity → cognitive image	0.305	*** (2.67e-10)	6.596	Supported
H4	Informational familiarity → affective image	0.203	*** (0.0000011)	4.996	Supported
H5	Cognitive image → overall image	0.354	*** (2.02e-10)	6.645	Supported
H6	Affective image → overall image	0.394	*** (4.36e-12)	7.294	Supported
H7	Overall image → satisfaction	0.506	*** (7.61e-19)	9.655	Supported
H8	Overall image → tourists’ loyalty	0.509	*** (6.86e-26)	11.875	Supported
H9	Cognitive image → affective image	0.529	*** (8.11e-28)	12.461	Supported
H10	Satisfaction → tourists’ loyalty	0.406	*** (1.50e-16)	8.889	Supported
H11	Overall image/satisfaction/tourists’ loyalty	0.001^a^	0.001^b^		Supported

^a^denotes direct relationship between overall image and tourists’ loyalty; ^b^denotes indirect relationship between overall image and tourists’ loyalty via satisfaction; ^c^denotes p-value < 0.001; ^d^denotes the actual p-value; *** denotes p -value < 0.001.

## Conclusion and Discussion

This study examined the relationship of the two dimensions of familiarity (experiential familiarity and informational familiarity) and two types of green hotels’ image (cognitive image and affective image) with the overall image, satisfaction, and loyalty to green hotels. The results indicated that experiential familiarity positively influences cognitive image (β = 0.438, *p* < 0.001) and affective image (β = 0.227, *p* < 0.001), in line with previous findings (Chi et al., 2020; Jebbouri et al., 2021). This shows that travelers’ past experience and knowledge about green hotels resulted in their favorable perceptions of green hotels’ attributes and characteristics. Thus, H1 and H2 were supported. The results demonstrated a significant positive correlation between informational familiarity and cognitive image (β = 0.305, *p* < 0.001) and affective image (β = 0.203, *p* < 0.001). These results correspond to previous studies that found that informational familiarity as an indirect experience can also influence travelers’ emotional response to green hotels (Soliman, 2019; Stylidis et al., 2020). Travelers can obtain information about green hotels through many different channels, such as brochures, websites, friends, newspapers, travel guidebooks, etc., that can lead to positive perceptions toward green hotels. Therefore, H3 and H4 were supported.

Cognitive image and affective image should be considered as separate factors affecting the overall image (Alcocer and Ruiz, 2019; Ragab et al., 2019). Cognitive image was a significant dimension that led to positive overall image (β = 0.354, *p* < 0.001), and affective image significantly influences overall image (β = 0.394, *p* < 0.001) toward green hotels. Travelers’ perceptions of green hotels’ attributes (e.g., reputations, value for money, and quality) and personal feelings significantly influence their overall evaluations of green hotels. Therefore, H5 and H6 were supported. Overall image (as a compilation which includes travelers’ knowledge, beliefs, and feelings toward green hotels) was found to have a direct positive correlation with satisfaction (β = 0.506, *p* < 0.001) and loyalty (β = 0.509, *p* < 0.001). These results are consistent with many previous studies which showed that overall image positively influenced tourists’ satisfaction and loyalty toward a destination (Králiková et al., 2020; Jebbouri et al., 2021). Travelers who have a more positive overall evaluation of green hotels tend to be more satisfied and more loyal to green hotels. Thus, H7 and H8 were supported. The results of this study also confirmed that cognitive image positively influences affective image (β = 0.529, *p* < 0.001). This means that travelers’ evaluation of affective characteristics and attributes of green hotels can be influenced by their cognitive components of knowledge and past experiences of green hotels. Thus, H9 was supported.

Based on previous studies, satisfaction should be considered as a crucial predictor of loyalty components (e.g., revisit intention, intention to recommend, word-of-mouth, etc.) (Casali et al., 2020; Stylidis et al., 2020). The results of this study show that there is a positive relationship between satisfaction and travelers’ loyalty toward green hotels (β = 0.406, *p* < 0.001), which means that travelers who are satisfied with green hotels’ attributes, characteristics, products, and services are more likely to revisit green hotels whenever they travel and are more likely to disseminate positive information about green hotels to others. In addition, the fact that satisfaction mediates the relationship between overall image and loyalty has been confirmed in many previous studies (Melo et al., 2017; Jebbouri et al., 2021). The mediation effect of satisfaction was supported based on the direct and indirect effects of the two-tailed significance test under bootstrapping at a 0.05 level. The results showed that the direct relationship between overall image and loyalty was found to be statistically significant (*p* = 0.001), whereas the indirect relationship between overall image and loyalty through satisfaction was also found to be statistically significant (*p* = 0.001). With that, satisfaction was found to have a partial mediation effect on the relationship between overall image and loyalty; thus, H11 was supported.

### Theoretical Contributions

First, familiarity is often defined as travelers’ past experience and knowledge of a destination (Chi et al., 2020), which is often used to predict their perceptions of image, satisfaction, and components of loyalty (e.g., revisit intention, word-of-mouth, etc.) (Kim et al., 2019; Casali et al., 2020). Nevertheless, familiarity cannot be equated to the single dimension of experiential familiarity but should also include informational familiarity. There are few studies that have investigated the relationship between the multidimensions of familiarity and green image and loyalty to patronizing green hotels. The study findings confirmed that both experiential familiarity and informational familiarity have a significant influence on the cognitive and affective image. Moreover, experiential familiarity displayed a slightly higher predictive capacity in influencing cognitive and affective image compared to informational familiarity. This indicates that travelers’ direct perceptions and knowledge exert more significant influence than indirect information acquired in determining the image of green hotels.

Second, travelers’ overall image of a specific destination is generally based on their knowledge and feelings toward the destination (Alcocer and Ruiz, 2019), and most researchers agreed that cognitive and affective images are the best predictors of the overall image, which lead to satisfaction and loyalty (Ragab et al., 2019; Králiková et al., 2020). However, there is scarce research on the relationship between green image and loyalty in the green hotel literature (Wang et al., 2018). The majority of previous studies have focused only on the cognitive attributes and characteristics, and the affective image was mostly neglected (Alcocer and Ruiz, 2019; Ragab et al., 2019). Kim and Richardson (2003) argued that the evaluation of the affective image of the destination can become more important than the evaluation of its objective attributes, but most researchers still believed that cognitive image represents a greater percentage of the general image than the affective aspects (Marine-Roig and Clavé, 2016). Kim et al. (2019) also stated that cognitive image provides a basis for overall image toward the destination, and therefore, it should occur before affective image develops. Even though many attitude theory researchers have long argued that the relationship between cognition and emotional components in attitude formation is bidirectional (Kim et al., 2019), there has been relatively little research on the interrelationship between the cognitive and affective image in tourism literature (Kim et al., 2019). The findings of this study findings confirmed that cognitive image and affective image have a separate influence on the overall image of green hotels, and furthermore, the affective image had more predictive power in determining travelers’ overall image when compared to the cognitive image. Nevertheless, the results also indicated that affective image is influenced by cognitive image.

Third, there is a lack of studies that explored the relationship between the green image, satisfaction, and loyalty in the green hotel literature (Wang et al., 2018), although green hotel studies seem to be a hot issue in the tourism literature. This study’s results showed that overall image significantly influenced satisfaction and loyalty toward green hotels. Meanwhile, satisfaction was found to mediate the relationship between overall image and loyalty. The study outcomes confirmed that overall image influenced travelers’ satisfaction, which subsequently leads to loyalty. These results provide the basis for future research in replicating such relationships in green hotel literature.

Finally, in the developing countries (e.g., China and India), consumers’ perception and recognition of green hotels are still low when compared to the western countries (Kasliwal and Agarwal, 2015; Wang, 2020). This study is the first to apply experiential familiarity, informational familiarity, cognitive image, and affective image in explaining travelers’ overall image, satisfaction, and loyalty toward patronizing green hotels in a developing country (i.e., China). The results suggest that researchers should implement the multidimensions of familiarity and image to predict travelers’ overall image, leading to satisfaction and loyalty in other developing countries to see if the findings are generalizable.

### Practical Implications

The results of this study provide a number of practical implications for green hotels. First, green hotels should provide more information on green hotels to potential customers in developing nations. Educating and promoting green hotels to existing and potential consumers to let them know what products and services can be provided by green hotels when compared to traditional hotels will be crucial in developing a favorable image for green hotels. Emphasizing the difference between green hotels’ attributes and traditional hotels’ attributes will be an important primary mission for such establishments, as experiential familiarity significantly influences cognitive and affective image. Meanwhile, advertising should be used as a tool to provide the necessary information to develop familiarity with green hotels. Green hotels can promote themselves through various channels, such as brochures, pamphlets, travel guidebooks, newspapers, and magazines, as well as using online platforms such as social media and websites.

To increase travelers’ brand knowledge and perceptions about green hotels, such establishments should publicize their green attributes and green business practices to potential customers, such as emphasizing how they practice water and energy conservation in order to enhance travelers’ perceptions of the intangible and abstract attributes of green hotels. Understanding how travelers perceive green hotels and identifying factors that affect their attitudes, feelings, and emotional response toward green hotels are important for creating an effective marketing strategy for green hotels.

### Limitations and Future Suggestions

There are certain limitations to the findings of this study. First, the study sample included only university students and does not represent the total population, which means that the results cannot be generalizable. Future studies should apply and validate the current framework by using a more representative sample and should test the interrelationship between cognitive and affective images. While this study focused on a broad concept of loyalty as a dependent variable, future studies may want to consider other possible components of loyalty, such as word-of-mouth, e-word-of-mouth, revisit intention, and recommendation to others. The model used in this study should be duplicated and extended with other theoretical frameworks in other locations to further confirm its validity and usefulness.

## Data Availability Statement

The raw data supporting the conclusions of this article will be made available by the authors, without undue reservation.

## Author Contributions

LW contributed to the design of the work, data collection, data analysis and interpretation, drafting the manuscript, and final approval of the version to be published. QZ contributed to the data collection and data analysis and interpretation. PW contributed to the data interpretation, drafting the manuscript, critical revision of the manuscript, and final approval of the version to be published. All authors contributed to the article and approved the submitted version.

## Conflict of Interest

The authors declare that the research was conducted in the absence of any commercial or financial relationships that could be construed as a potential conflict of interest.

## Publisher’s Note

All claims expressed in this article are solely those of the authors and do not necessarily represent those of their affiliated organizations, or those of the publisher, the editors and the reviewers. Any product that may be evaluated in this article, or claim that may be made by its manufacturer, is not guaranteed or endorsed by the publisher.

## Footnotes

1
www.wenjuan.com
